# Commentary: Likelihood Ratio as Weight of Forensic Evidence: A Closer Look

**DOI:** 10.3389/fgene.2018.00224

**Published:** 2018-06-22

**Authors:** Colin Aitken, Anders Nordgaard, Franco Taroni, Alex Biedermann

**Affiliations:** ^1^School of Mathematics, University of Edinburgh, Edinburgh, United Kingdom; ^2^National Forensic Centre (Sweden), Linköping, Sweden; ^3^School of Criminal Justice, Université de Lausanne, Lausanne, Switzerland

**Keywords:** likelihood ratio, value of evidence, forensic science, logarithm, forensic reporting

A recent article (Lund and Iyer, 2017) provides, in the words of its title, a closer look at the likelihood ratio as the weight of forensic evidence. This note comments critically on two aspects of the article.

The first aspect concerns two related statements. In the abstract the statement is made that “[W]e find the likelihood ratio paradigm to be unsupported by arguments of Bayesian decision theory, which applies only to personal decision making and not to the transport of information from an expert to a separate decision maker.” The idea presented in this statement of lack of support for the likelihood ratio as a means of transport of information is repeated in the conclusion where it is stated that “… we hope the forensic science community comes to view the *LR* as one *possible*, not normative or necessarily optimum, tool for communicating to DMs (decision makers)” (Lund and Iyer's emphasis). Despite this opinion of these authors, it was shown many years ago by I.J.Good in two brief notes in the *Journal of Statistical Computation and Simulation* (Good, 1989a,b) repeated in Good (1991) and in Aitken and Taroni (2004) that, with some very reasonable assumptions, the assessment of uncertainty inherent in the evaluation of evidence leads inevitably to the likelihood ratio as the only way in which this can be done.

In order to show that the likelihood ratio is the only way to evaluate evidence, it is necessary to introduce some mathematical notation. This is a device to ease the presentation of the argument. The argument could be made verbally but would be lengthy and more difficult to follow. Consider evidence *E* which it is desired to evaluate in the context of two mutually exclusive propositions *H*_*p*_ and *H*_*d*_. Denote the value of the evidence by *V*. Of course, this statement makes the implicit assumption that evidence has a value that can be measured. The value will depend on background information *I*. Four and only four factors have been introduced, *E, H*_*p*_, *H*_*d*_ and *I*. Thus, *V* is a function of these four factors, *V* = *f*(*E, H*_*p*_, *H*_*d*_, *I*). There is uncertainty about *E*, so it should be analyzed probabilistically. Use of the argument of conditional probability leads to *f*(*E* ∣ *H*_*p*_, *H*_*d*_, *I*)*f*(*H*_*p*_, *H*_*d*_, *I*), rather than forms such as *f*(*H*_*p*_ ∣ *H*_*d*_, *E, I*) or variants of it. The expression *f*(*H*_*p*_, *H*_*d*_, *I*) does not involve the evidence, which reduces considerations further to *f*(*E* ∣ *H*_*p*_, *H*_*d*_, *I*). Propositions *H*_*p*_ and *H*_*d*_ are mutually exclusive so if *E* is to be a function of both *H*_*p*_ and *H*_*d*_ then *f*(*E* ∣ *H*_*p*_, *H*_*d*_, *I*) is a combination of two functions, one that involves *H*_*p*_ and not *H*_*d*_ and one that involves *H*_*d*_ and not *H*_*p*_. Value may thus be expressed as a function of the probabilities of *E* given *H*_*p*_ (and *I*) and of *E* given *H*_*d*_ (and *I*). Again, this makes implicit assumptions, namely that there is a probability that can be associated with evidence and that is dependent on a proposition and background information. For ease of notation explicit mention of *I* will be omitted from notation in what follows.

Let *x* = Pr(*E* ∣ *H*_*p*_) and *y* = Pr(*E* ∣ *H*_*d*_). The assumption that *V* is a function only of these probabilities can be represented mathematically as

V=f(x,y)

for some function *f*.

Now, consider another piece of evidence *T* which is irrelevant to *E*, to *H*_*p*_ and to *H*_*d*_. Irrelevance is taken in the probabilistic context to be equivalent to independence so that *T* may be taken to be independent of *E*, of *H*_*p*_ and of *H*_*d*_. It is then permissible for Pr(*T*) to be given notation which does not refer to any of *E, H*_*p*_ or *H*_*d*_. Thus, let Pr(*T*) be denoted by θ. Then

$$\begin{array}{l} \mathrm{Pr}(E,T|H_{p})\\ \;=\mathrm{Pr}(E|H_{p})\mathrm{Pr}(T|H_{p})\text{ by the independence of }E\text{ and }T\\ \;=\mathrm{Pr}(E|H_{p})\mathrm{Pr}(T)\text{ by the independence of }T\text{ and }H_{p}\\ \;=x\theta . \end{array}$$

Similarly,

$$\mathrm{Pr}\left(E,T|H_{d}\right)=y\theta .$$

The value of (*E, T*) is *f*(θ*x*, θ*y*) by the definition of *f*. However, evidence *T* is irrelevant and has no effect on the value of evidence *E*. Thus, the value of the combined evidence (*E, T*), *f*(θ*x*, θ*y*), is equal to the value *V* of *E, f*(*x, y*), and

$$\begin{array}{l} V=f\left(x,y\right)=f\left(\theta x,\theta y\right) \end{array}$$

for all θ in the interval [0,1] of possible values of Pr(*T*).

The only class of functions of (*x, y*) for which this can be said to be the case is the class which are functions of *x*/*y* or

$$\mathrm{Pr}\left(E|H_{p}\right)/\mathrm{Pr}\left(E|H_{d}\right)$$

which is the likelihood ratio. Hence the value *V* of evidence has to be a function of the likelihood ratio. Lund and Iyer wish the forensic community to view the likelihood ratio as one possible tool for communication with decision makers. We hope that we have shown here through the argument of Good that it is the only logically admissible form of evaluation. Incidentally, note that no recourse has been made to arguments of Bayesian decision theory. The support of these arguments for the likelihood ratio paradigm, as suggested in the abstract, is not necessary.

The second aspect is minor and concerns a definition. The concept of weight of evidence is an old idea. The term *weight of evidence* for the logarithm of the likelihood ratio was given by Charles Sanders Peirce (Peirce, 1878). It is not the likelihood ratio that should be referred to as the weight of evidence as is done in the title of the article. It is better to refer to the likelihood ratio as the *value* of the evidence and its logarithm as the weight of the evidence. The logarithm of the likelihood ratio has the pleasingly intuitive operation of additivity when converting the logarithm of the prior odds in favor of a proposition to the logarithm of the posterior odds in favor of the proposition.

(1) $$\begin{array}{r} \log\{\frac{\mathrm{Pr}\left(H_{p}|E\right)}{\mathrm{Pr}\left(H_{d}|E\right)}\}=\log\{\frac{\mathrm{Pr}\left(E|H_{p}\right)}{\mathrm{Pr}\left(E|H_{d}\right)}\}+\log\{\frac{\mathrm{Pr}\left(H_{p}\right)}{\mathrm{Pr}\left(H_{d}\right)}\}. \end{array}$$

When considering the scales of justice it is the logarithm of the probabilities of the evidence given each of the two competing propositions that should be put in the scales, not the probabilities. Equation (1) can be rewritten as

$$\begin{array}{l} \log\left\{\mathrm{Pr}\left(H_{p}|E\right)\right\}-\log\left\{\mathrm{Pr}\left(H_{d}|E\right)\right\}=\\ \log\left\{\mathrm{Pr}\left(E|H_{p}\right)\right\}-\log\left\{\mathrm{Pr}\left(E|H_{d}\right)\right\}+\log\left\{\mathrm{Pr}\left(H_{p}\right)\right\}-\log\left\{\mathrm{Pr}\left(H_{d}\right)\right\}\\ =\left[\log\left\{\mathrm{Pr}\left(E|H_{p}\right)\right\}+\log\left\{\mathrm{Pr}\left(H_{p}\right)\right\}\right]-\left[\log\left\{\mathrm{Pr}\left(E|H_{d}\right)\right\}+\log\left\{\mathrm{Pr}\left(H_{d}\right)\right\}\right] \end{array}$$

Expressions to the left of the negative sign in the last line are associated with one pan in the scales, expressions to the right with the other pan. Thus log(Pr(*E* ∣ *H*_*p*_)) is added to the prior log probability for *H*_*p*_ in one scale and log(Pr(*E* ∣ *H*_*d*_)) is added to the prior log probability for *H*_*d*_ in the other scale. The difference in the sums of the two pairs of log probabilities is a more intuitive characteristic of the evidence to which the term *weight* may be applied than the ratio of the probabilities of the evidence given the respective propositions.

## Author contributions

CA drafted this commentary which results from equal and direct intellectual contributions of all listed authors.

### Conflict of interest statement

The authors declare that the research was conducted in the absence of any commercial or financial relationships that could be construed as a potential conflict of interest.

## References

[B1] Aitken C. G. G. Taroni F. (2004). Statistics and the Evaluation of Evidence for Forensic Scientists, 2nd Edn. Chichester: John Wiley and Sons Ltd.

[B2] Good I. J. (1989a). C312: yet another argument for the explication of weight of evidence. J. Stat. Comput. Simul. 31, 58–59. 10.1080/00949658908811115

[B3] Good I. J. (1989b). C319: weight of evidence and a compelling metaprinciple. J. Stat. Comput. Simul. 31, 121–123. 10.1080/00949658908811131

[B4] Good I. J. (1991). Weight of evidence and the Bayesian likelihood ratio in The Use of Statistics in Forensic Science, eds Aitken C. G. G. Stoney D. A. (Chichester: Ellis Horwood), 85–106.

[B5] Lund S. P. Iyer H. (2017). Likelihood ratio as weight of forensic evi-dence: a closer look. J. Res. Natl. Inst. Stand. Technol. 122:27 10.6028/jres.122.027 PMC7339646 34877093

[B6] Peirce C. S. (1878). The probability of induction, in The World of Mathematics, 1956, Vol. 2, ed Newman J. R. (New York, NY: Simon Schuster), 1341–1354.

